# A noval approach based on TCN-LSTM network for predicting waterlogging depth with waterlogging monitoring station

**DOI:** 10.1371/journal.pone.0286821

**Published:** 2023-10-12

**Authors:** Jinliang Yao, Zhipeng Cai, Zheng Qian, Bing Yang

**Affiliations:** 1 Hangzhou Dianzi University, Hangzhou, China; 2 Ningbo Meteorological Service Center, Ningbo, China; 3 Key Laboratory of Brain Machine Collaborative Intelligence of Zhejiang Province, Hangzhou, China; Taipei Medical University, TAIWAN

## Abstract

As a result of climate change and rapid urbanization, urban waterlogging commonly caused by rainstorm, is becoming more frequent and more severe in developing countries. Urban waterlogging sometimes results in significant financial losses as well as human casualties. Accurate waterlogging depth prediction is critical for early warning system and emergency response. However, the existing hydrological models need to obtain more abundant hydrological data, and the model construction is complicated. The waterlogging depth prediction technology based on object detection model are highly dependent on image data. To solve the above problem, we propose a novel approach based on Temporal Convolutional Networks and Long Short-Term Memory networks to predicting urban waterlogging depth with Waterlogging Monitoring Station. The difficulty of data acquisition is small though Waterlogging Monitoring Station and TCN-LSTM model can be used to predict timely waterlogging depth. Waterlogging Monitoring Station is developed which integrates an automatic rain gauge and a water gauge. The rainfall and waterlogging depth can be obtained by periodic sampling at some areas with Waterlogging Monitoring Station. Precise hydrological data such as waterlogging depth and rainfall collected by Waterlogging Monitoring Station are used as training samples. Then training samples are used to train TCN-LSTM model, and finally a model with good prediction effect is obtained. The experimental results show that the difficulty of data acquisition is small, the complexity is low and the proposed TCN-LSTM hybrid model can properly predict the waterlogging depth of the current regional. There is no need for high dependence on image data. Meanwhile, compared with machine learning model and RNN model, TCN-LSTM model has higher prediction accuracy for time series data. Overall, the low-cost method proposed in this study can be used to obtain timely waterlogging warning information, and enhance the possibility of using existing social networks and traffic surveillance video systems to perform opportunistic waterlogging sensing.

## 1 Introduction

In recent decades, with increasing of urban spatial scale and climate change, the occurrence of urban waterlogging has become more frequent and more serious due to heavy rainfall in developing countries. Urban waterlogging results in traffic jams and even paralysis. The severe ones cause great economic loss and heavy casualty. For example, extremely heavy rainstorm caused severe waterlogging in Beijing, China, on July 21, 2012. This rainstorm resulted in 79 deaths and the direct economic loss of approximately 1.80 billion USD [1]. Therefore, it is of great importance to create an urban waterlogging monitoring and early warning system for reducing loss of life or injury, property damage, and economic disruption in future disasters. This system is able to obtain the real-time waterlogging depth and rainfall at some waterlogging prone areas. It is helpful to assess the risk of waterlogging and generate early warning information. It can further improve the response-ability of urban administration in facing waterlogging disasters. The critical component of early warning system is obtaining real-time waterlogging depth and accurately predicting waterlogging depth based on real-time information.

Currently, there are three main ways to obtain urban waterlogging depth. One way is based on satellite remote sensing image [2]. For example, Tran et al [3] obtained waterlogging depth through Google search engine combined with satellite remote sensing image technology. The second way is based on computer vision [4–6]. Huang et al. [4] estimated waterlogging depth using images from social networks and traffic surveillance video systems. The third way is based on water level sensors [7]. The obtained waterlogging depth based on the first two ways is low accuary, non-real time, and high computation complexity. In this study, Waterlogging Monitoring Station(WMS) was used to obtain real-time and high accurate urban waterlogging depth. WMS is a real-time data acquisition device that integrates sensors, the Internet of Things and cloud storage. The sensors of WMS include an automatic rain gauge and a water gauge. Commonly, WMSes are deployed at specific spots where is susceptible to waterlogging. The proposed system with WMS can obtain the rainfall and waterlogging depth of specific spots.

There are some related methods for predicting water level. The common methods of water level prediction are based on statistical machine learning. Zhang et al. [8] used wavelet analysis and Artificial Fuzzy Neural Network to predict the water level of Poyang Lake. Daliakopoulos et al. [9] proposed Feed Forward Neural Network(FFNN) for forcasting groundwater level. With the rapid development of deep learning technology, deep learning regression models have gradually been applied to water level prediction. Chen et al. [10] applied the LSTM model for water level prediction and found that the model is better than support vector machine. Sz et al. [11] presented a joint model of FFNN and LSTM to predict lake water level. They found that LSTM and FFNN models outperform nonlinear regression, exponential smoothing, and Autoregressive Rntegrated Moving Average models. Phan and Hoai [12] proposed a hybrid approach that takes advantages of linear and nonlinear models. The proposed method combines statistical machine learning algorithms and Autoregressive Integrated Moving Average for forecasting water level. Barbosa et al. [13] used data generated by a regionalized climate model as input to a simple hydrological model and a one-dimensional vertical hydrodynamic model. The two models were used to predict the potential changes in the Itupararanga reservoir, SaoPaulo, Brazil, in an exemplary time period (2028–2030). In addition, a few of scholars proposed Artificial Neural Network (ANN) and an intelligent fuzzy regression algorithm for the freight transportation prediction [14]. Pan et al. [15] built a CNN-GRU model in which the GRU learns the changing trend of water level, and the CNN learns the spatial correlation among water level data ob-served from adjacent water stations. Huang [16] proposed TCN (temporal convolutional network) to predict dissolved water quality. The prediction accuracy based on TCN reached up to 91.91%. The time costs of training model and prediction are reduced by an average of 64.92% and 7.24%,respectively. Hrnjica and Bonacci [17] use LSTM to forcast the water level of Vrana Lake. However, the problem they want to solve is a long term prediction in a large-scale water area, such as lake and river.

Encouragingly, more and more scholars have begun to study the waterlogging depth prediction recently. Zhang et al. [18] proposed a multi-strategy-mode-waterlogging-prediction framework for predicting waterlogging depth based on time series prediction and machine learning regression algorithm. Wu et al. [19] proposed a regression model constructed with deep learning algorithm, named Gradient Boosting Decision Tree(GBDT), to predict the depth of urban flooded areas. Wei et al. [20] established an early warning system that can predict the whole rainfall process according to the rainfall curve of the first 20 minutes based on coupling RBF-NARX neural networks. And this early warning system is quite effective in avoiding and reducing the losses from waterlogging. Jing et al. [4] used the Mask R-CNN network for detecting ties from surveillance video and then calculate the waterlogging depth by the size of ties. But this method is based on the assumption that vehicles will pass through the waterlogged road, this condition is difficult to meet. Jingchao et al. [6], in view of the difficulty of image data acquisition, further optimized the image synthesis technology combined with CNN network to reduce the difficulty of image acquisition. Due to the high complexity and low prediction accuracy of hydrological models, some researchers began to introduce deep learning models to optimize them. Yuanyuan et al. [21] proposed the LSTM-Numerical Model (LSTM-NM) and introduced the LSTM model to simplify the hydrological model. Even more some researchers have abandoned hydrological models altogether, Yuchen et al. [22] proposed a machine learning model—AutoML which used the data of historical rainfall and waterlogging depth as training samples. AutoML is used to predict waterlogging depth data.

However, among the methods proposed in the present research, images detection models (such as Mask R-CNN) are highly dependent on image data, and the hydrological model needs abundant hydrological data. In this paper, we proposed a timely waterlogging depth prediction method based on WMS and TCN-LSTM model. The primary features of our method are as follows.

1. WMS is a new type of waterlogging depth and rainfall collection sensor. It integrates an automatic rain gauge and a water gauge. The rainfall and waterlogging depth at the installation position are obtained by periodic sampling of WMS and transferred to server application by the Internet of Things. The data of rainfall and waterlogging depth is easily accessible, accurate, timely, sequential and reliable.
2. In this paper, we focus the real-time capability of early warning system based on the remote sensing data provided by WMS, such as waterlogging depth and rainfall. The proposed method is able to predict the waterlogging depth at the particular areas in ten or thirty minutes based on the historical data.
3. Dependencies in sequence prediction problems. Based on the time series data obtained from WMS, we proposed a TCN-LSTM hybrid model, waterlogging depth and rainfall collected by WMS are used as training samples. Then training samples are used to train TCN-LSTM network, and finally a model for predicting waterlogging depth is obtained. The hybrid model proposed in this study can be used to obtain timely waterlogging warning information, and enhance the possibility of using existing social networks and traffic surveillance video systems to perform opportunistic waterlogging sensing.

The structure of this paper is as follows. The second part presents our used data and its acquisition method. The third part introduces our proposed waterlogging depth prediction method. The fourth part is the experimental results and discussion. Finally, the last part summarizes our work.

## 2 Data acquisition

WMS integrates an electronic water gauge, a rainfall sensor and the Internet of Things which is used to transmit data online. WMS is used to obtain real-time and high accurate urban waterlogging depth data. These WMSes are installed at some areas that prone to waterlogging. Table 1 shows the obtained time series data which include timestamp, rainfall and waterlogging depth. The sampling period is settable. In our experiments, the sampling period is set to 5 minutes which is timely and effective.

**Table 1:** Waterlogging depth data come from WMS.

Time	Waterlogging Depth(mm)	Rainfall(cm)
2018–09–19 07:28:46	10	0.2
2018–09–19 07:23:46	0	0.8
2018–09–19 07:18:46	30	0
2018–09–19 07:13:46	70	0
2018–09–19 07:08:46	80	0
2018–09–19 07:03:46	90	0.6
2018–09–19 06:58:46	40	3
2018–09–19 06:53:46	22	5
2018–09–19 06:48:46	22	0.8
2018–09–19 06:43:46	10	0.2
2018–09–19 06:38:46	10	3.4
2018–09–19 06:33:46	10	4.8

**Fig 1:**
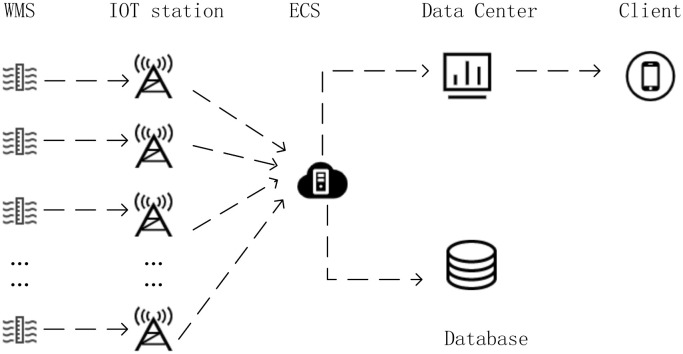
The process of data acquisition and transmission. BS: IOT Base Station.

The critical components of WMS are electronic water gauge, single bucket rain gauge, solar panel, a data acquisition unit, communication system and particular battery. Electronic water gauge is a type of electronic water level sensor that uses a contact sensor to track the rise and fall of water levels. The water gauge is readable to ±0.5 cm. The tipping bucket rain sensor is a hydrological and meteorological instrument used to measure natural rainfall. It converts collected data into digital information in the form of a switching value to transmit, process, record, and display information. The accuracy of single tipping bucket rain gauge is 0.2mm. Its measurement error is less than 8%. The solar-powered WMS integrates an electronic water gauge and a tipping bucket rainfall sensor into one unit. The waterlogging depth and rainfall data is periodically sent to server using wireless Internet of things technology. As shown in Fig 1, the early warning system as an application obtains each spot waterlogging depth and rainfall data by calling a interface.

Currently, some WMSes have been deployed in Ningbo City. The climate of Ningbo is temperate, with relatively cold winters and hot, muggy, and rainy summers. Ningbo is a metropolis located in eastern China, in Zhejiang, on the East China Sea coast. The rainfall of Ningbo is quite abundant, and amounts to 1,400 millimeters per year. Nevertheless, rainfall is below 100 mm per month from October to February. Summer, a season when the rains occur mainly in the form of showers or thunderstorms, is the rainiest season, especially in June. WMSes are primarily installed on culverts, tunnels, green belts, highways, and etc. These WMSes have been deployed for two years, and obtained a lot of data.

## 3 Methodology

Waterlogging depth prediction is the time series prediction problem. In general, the time series prediction problem is expressed by the Eq 1.

$$\begin{array}{c} y(t+d)=f(x(t),\;x(t-1),\;\ldots ,\;x(t-m+1)) \end{array}$$ (1)

As the Eq 1 shows, time series data is a sequence of vectors, x(t), t = 0, 1, …, where t represents elapsed time. x(t) represents a vector which consists of waterlogging depth and rainfall data at t; x is sampled to obtain a series of discrete data points, equally spaced in time. In this case, sample time is set to 5 minutes. y is a value which varies continuously with t, and represents the predicted waterlogging depth at time (t + d) in the future. Formally, the objective of this work is to find a function f to obtain an estimate of y at time (t + d) from x(t),x(t − 1),…,x(t − m + 1).

**Fig 2:**
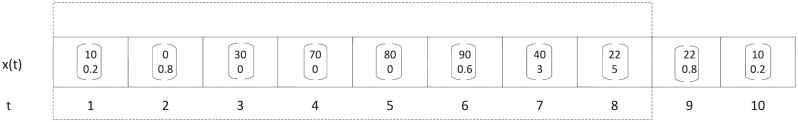
Initial state of sliding window. x(t) represents a vector consisted of waterlogging depth and rainfall at t.

**Fig 3:**
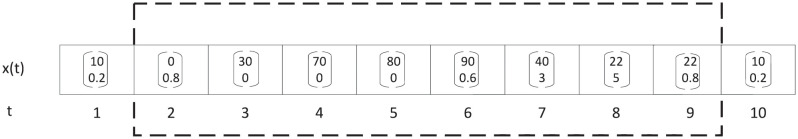
Process of sliding window. x(t) represents a vector consisted of waterlogging depth and rainfall at t.

In this paper, function f is fitted by the TCN-LSTM model to predict the future waterlogging depth. TCN has good feature extraction ability. LSTM is a special kind of recurrent neural network capable of handling long-term dependencies, and can be used to model univariate time series forecasting problems. We used the sliding window to get the N-tuple by sliding over the full training set. The size of sliding window is 8, that is, the rainfall and waterlogging depth of the past 40 minutes from (t—7) to t are selected as the input vector to predict the waterlogging depth at t + d. Fig 2 shows the initial state of sliding window Fig 3 shows the process of sliding window.

### 3.1 TCN model

TCN [23] is a special 1-D full CNN, as a variant of the CNN, TCN performs better in time series prediction than the RNN and LSTM. It is more suitable for processing sequential data with large receptive fields and temporality [24]. The design idea of TCN is based on two principles: one is that the length of the output is the same as that of the input, and the other is that there can be no leakage from the future into the past. The TCN module integrates fully convolutional network (FCN), causal convolution, dilation convolution, and residual blocks, and has a high parallel processing capability, stable gradient, and flexible perception field that LSTM lacks.

The application of TCN in time series data prediction is derived from this article: An Empirical Evaluation of Generic Convolutional and Recurrent Networks for Sequence Modeling [23]. In this paper, Shaojie Bai, J. Zico Kolter and Vladlen Koltun, three authors proposed the Time Convolutional Network and conducted experiments on datasets(such as MNIST, Word-level PTB and Char-level PTB) obtained in multiple different fields. The results show that TCN is more accurate and universal in processing long sequence data. Compared with traditional RNN, TCN has more flexible receptive field size, good parallelism, and stable gradient. This is also why TCN is more efficient than RNN. With the publication of this paper, the research and application of TCN in time series data prediction is increasing. Researchers has started to combine with the traditional RNN, token advantages of each network to achieve better results. For example, Wei Cheng et al. [25] proposed a TCN-CBAM model by combining TCN with an embedded convolution block attention module (CBAM). This model is superior to LSTM, CNN-LSTM and TCN models in predicting classical systems (Chen system, Lorenz system and sunspots). Wenshu Li et al. [26] for the first time combined TCN and LSTM model to predict the concentration of dissolved oxygen in aquaculture, and the effect was better than the traditional RNN model. These researches further confirmed the effect of TCN in this field.

These researches further proves the reliability and effectiveness of TCN in time series data prediction. Therefore, based on the above research results, this paper introduced TCN and combined with traditional LSTM to carry out experiments and demonstration in the field of urban waterlogging depth prediction.

**Fig 4:**
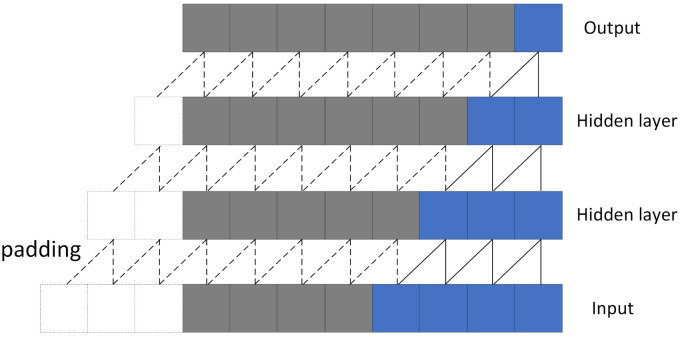
A casual convolution with filter kernel size k = 2.

Causal convolution, a strict time-constrained scheme, indicates that output at time t only depends on elements from time t and earlier in the previous layer. Different from the traditional convolutional neural network, for the prediction of time t, only the observed sequence (*x*_1_, *x*_2_, …, *x*_(*t*−1)_, *x*_*t*_) will be used instead of the future information in TCN. As shown in Eq 2. However, causal convolutions need to increase the number of layers to capture more information, thus making the network deeper and more complex. For the time series prediction tasks that need to acquire a long history, the dilated convolution is used to increase the perception field of the network. Fig 4 shows an example of causal convolution.

$$\begin{array}{c} p\left(x\right)=\;\prod_{t=1}^{T}\;p(x_{t}|x_{1},x_{2},{\ldots ,x}_{t-1}) \end{array}$$ (2)

**Fig 5:**
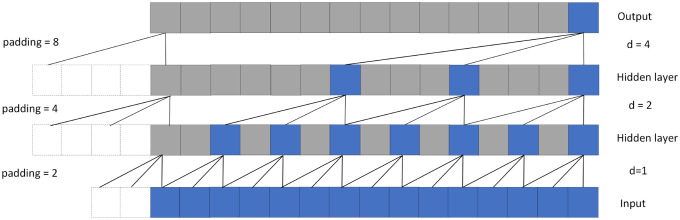
A dilated casual convolution with dilated factors d = 1,2,4 and filter kernel size k = 3.

Dilated convolution obtains larger perception field through interval sampling, making up for the defects of causal convolution. For a 1-D sequence input x ∈ Rn and a filter f: {0, …, *k* − 1} → R, the dilated convolution operation F on element s of the sequence is defined as

$$\begin{array}{c} F\left(s\right)=(x*{}_{d}^{}f)\left(s\right)=\sum_{i=0}^{k-1}\;f(i)\cdot x_{s-d\cdot i} \end{array}$$ (3)

where d is the dilation factor, k is the filter size, and s − d • i accounts for the direction of the past. Dilation is thus equivalent to introduce a fixed step between every two adjacent filter taps. When d = 1, a dilated convolution reduces to a regular convolution. A larger dilation factor enables an output at the top level to represent a wider range of inputs, thus effectively expanding the perception field of ConvNet. According to the Eq 3, increasing the dilation factor d and the convolution kernel size k could increase the receptive field of TCN. In general, the dilation factor d increases exponentially as the network deepens. Fig 5 shows an example of dilation convolutions. In the figure, the dilation factors d = 1, 2 4 and the convolution kernel size k = 3.

The residual block is used to decrease the computing complex, and handle the problem of gradient explosion and gradient disappearance. The equation of residual block can be expressed as Eq 4.

$$\begin{array}{c} x_{l+1}=h(x_{l})+F(x_{l}\;,W_{l}) \end{array}$$ (4)

**Fig 6:**
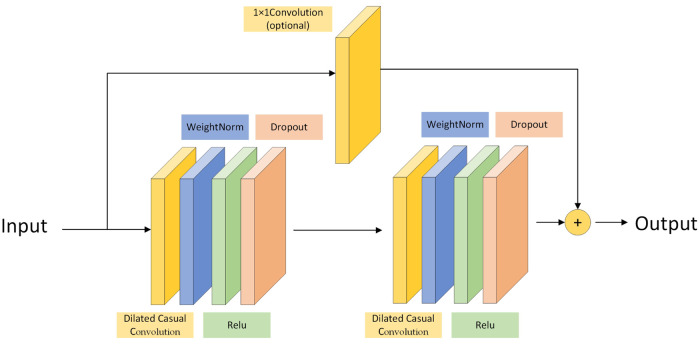
The structure of TCN residual block.

Where F(*x*_*l*_, *W*_*l*_) is the residual part, and *W*_*l*_ is the weight matrix. h(*x*_*l*_) is the direct mapping part, including 1 × 1 convolution operation. *x*_*l*_ is the input of the residual block, and *x*_*l*+1_ is the output of the residual block. Fig 6 shows the residual block structure in TCN. The residual block has two layers, including dilated causal convolution, weight normalization, non-linearity(Relu), and dropout layer for regularization. If the input and output of residual have different dimensions, it is usually solved by adding a 1 × 1 convolution. The residual network adds the identity map of cross layer connection. It is beneficial for the stability of TCN to build deeper network structure.

### 3.2 LSTM model

For a given time series $\hat{X}=\{{\hat{x}}_{1},\ldots ,{\hat{x}}_{t},\ldots ,{\hat{x}}_{T}\}$ after TCN’s feature extraction, the mapping from ${\hat{x}}_{t}$ to *h*_*t*_ at time t should be learn, which is obtained as

$$\begin{array}{c} {\;h}_{t}=f_{1}(h_{t-1},\;{\hat{x}}_{t}) \end{array}$$ (5)

where *h*_*t*−1_ and *h*_*t*_ are hidden states of LSTM at time t − 1 and t, respectively. The nonlinear function *f*_1_ is an LSTM unit.

**Fig 7:**
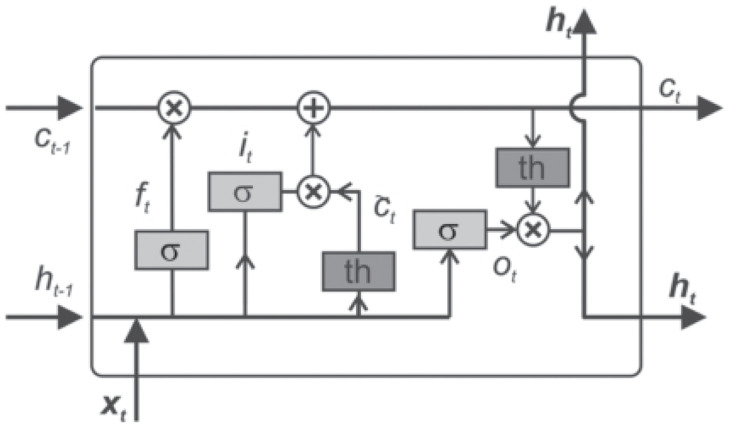
A common LSTM cell unit.

LSTM [27] is a type of recurrent neural network which is capable of handling long-term dependencies. As shown in Fig 7, a common LSTM cell unit is composed of an input gate, an output gate and a forget gate. The forget gate is adopted to control how much information of the cell state is forgotten at the previous time. The input gate is adopted to control how much information of the input is added to the cell state at the current time. The output gate is adopted to control how much information of the cell state is adopted as the output at current time. The calculation process of LSTM cell unit from the input to the output is expressed as the following equations.

$$\begin{array}{c} f_{t}=\sigma (W_{f}[h_{t-1},\;{\hat{x}}_{t}]+\;b_{f}) \end{array}$$ (6)

$$\begin{array}{c} i_{t}=\sigma (W_{i}[h_{t-1},\;{\hat{x}}_{t}]+\;b_{i}) \end{array}$$ (7)

$$\begin{array}{c} {\tilde{c}}_{t}=tanh(W_{c}[h_{t-1},\;{\hat{x}}_{t}]+\;b_{c}) \end{array}$$ (8)

$$\begin{array}{c} c_{t}=f_{t}\;⊙{\;c}_{t-1}+i_{t\;}⊙{\tilde{\;c}}_{t} \end{array}$$ (9)

$$\begin{array}{c} o_{t}=\sigma (W_{o}[h_{t-1},\;{\hat{x}}_{t}]+\;b_{o}) \end{array}$$ (10)

$$\begin{array}{c} h_{t}=o_{t}\;⊙\;tanh(c_{t}) \end{array}$$ (11)

Where f, i, o and ${\tilde{c}}_{t}$ represent forget gate, input gate, output gate, and candidate vector, respectively, *W*_*f*_, *W*_*i*_, *W*_*o*_ and *W*_*c*_ denote weight matrices. *b*_*f*_, *b*_*i*_, *b*_*o*_ and *b*_*c*_ are bias vectors. *σ*(⋅) and tanh(⋅) denote the sigmoid and hyperbolic tangent functions, respectively. c represents cell state.

### 3.3 TCN-LSTM model

**Fig 8:**
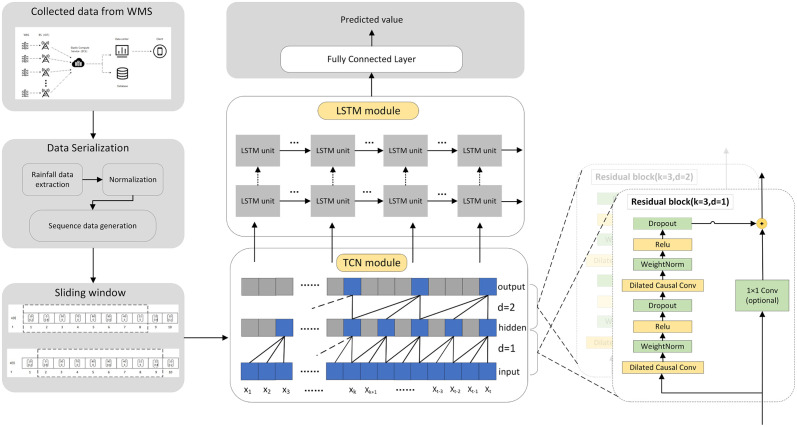
The architecture of TCN-LSTM model. The TCN module can be replaced with CNN model. The LSTM module can be replaced with RNN or GRU to verify the effectiveness of different prediction models.

The model architecture based on TCN-LSTM module proposed in this paper is shown in Fig 8. The proposed model consists of an input layer, two TCN modules, two LSTM layers, a fully connected layer and an output layer. After data preprocessing, we employed sliding window over the input sequence to get a set of N-tuples as input data. The equation can be expressed as Eq 12

$$\begin{array}{c} x\left(t\right),\;x(t-1),\;\ldots ,\;x(t-m+1)=S_{sliding\;window}(x,m,t) \end{array}$$ (12)

where S denotes sliding window, as given in Fig 3. m and t denote the window size and current time, respectively. The input layer is used to feed the *x*_*t*_, *x*_*t*−1_,…,*x*_*t*−*m*+1_ into the TCN. The two TCN modules are the core of the TCN-LSTM model. TCN has good feature extraction ability for time series data. We perform feature extraction over input data through TCN, which consists of two residual blocks. The first one is consisted of two causal dilated convolution layers with a kernel size of 3, a dilation factor of 1, and the filter number of 10. The second one has a dilation factor of 2, and the remaining parameters are the same as those of the first one. The output of the TCN layers is then reshaped and fed to the LSTM layers to extract the long-term dependence in a sequence. LSTM is able to capture the short-term and long-term dependencies, and is widely applied in time series prediction problems. Then the fully connected layer (FC) is introduced to reduce the dimension of data. Finally, the output layer outputs the predicted waterlogging depth. TCN has an expanded causal convolution structure and outstanding feature extraction ability, it can fuse original features to obtain high-dimensional abstract features, which enhances feature information mining. LSTM has strong ability of time series data prediction. By combining TCN and LSTM, data features are extracted through TCN and then input into LSTM, the processing efficiency of LSTM memory unit is faster and more effectively.

The TCN module in this study uses Relu activation function, based on its characteristics: 1. When the input is positive, there is no gradient saturation problem. 2. Fast calculation speed. Relu function only has linear relationship, so its calculation speed is faster than Sigmoid function and Tanh function. 3. For the Dead Relu problem, when the input is negative, Relu will completely fail. In this study, since the waterlogging depth data itself does not have negative values, so there is no Dead Relu problem. The researchers also used the Relu activation function [28], with great success. LeakyReLU activation function is used [25], which is a variant Relu activation function, mainly to effectively solve the problem that neurons can-not learn when the Relu function input is negative, namely the Dead Relu problem.

In the training process of the hybrid model proposed in this paper, multiple parameters need to be set, including the expansion factor d of the TCN, the convolution kernel k, the number of LSTM neurons, the learning rate, the batch size and the overfitting parameters. The setting of these parameters will directly affect the results of model training.

In this study, if the length of the input sequence is to be larger, the size of the sliding window can be adjusted, and the size of the TCN expansion factor d and the convolution kernel k can be increased at the same time to obtain a larger input receptive field. However, since the correlation between the rainfall at the earlier time and the rainfall at the future time is already weak, the best input sequence length M = 8. Trial and error method is introduced to adjust the size of d and k, and finally d = 4, k = 3 are best. For the selection of learning rate and overfitting parameters, this study selected the optimal value through grid search algorithm. We selected a group of learning rate parameters and a group of overfitting parameters, built models according to each coordinate, and selected the learning rate and overfitting parameters with the best model effect as the optimal solution.

## 4 Results and discussion

### 4.1 Dataset and data preprocessing

There are different terrain and drainage conditions at different locations where WMS deployed. We need to train different models for different locations. The time series data of different WMSes is collected, which include Huaihe Road, Dagangzhong Road, Donghe Road, and so on. The dataset is consisted of 10625 rows of raw data collected in Ningbo from August 17, 2018 to October 18, 2019. Each row data includes timestamp, rainfall and waterlogging depth. The sampling period is set to 5 minutes to meet the requirement of prediction accuracy and real-time performance. The dataset was splited in 90:10 ratio i.e. 90% of the data is used for training the model while 10% is used for testing the model. We mainly chose two data sets: Huaihe Road and Danggangzhong Road for training and test our model according to the number of valid data. The following is the detailed description of these two data sets, and the information of these two data sets is shown in Table 2:

**Table 2:** The information of two data sets.

Dataset	HuaiheRoad Dataset	DagangzhongRoad Dataset
Total data	1493	1597
Numerical range	0–320	0–360
Time	2018.8.17–2018.9.19	2018.8.17–2018.9.19
Time Intervals	5 minutes	5 minutes
Train/Test	1344/149	1438/159

The scale and quality of the data is important for a deep regression analysis model. The probability of rainy day in Ningbo is less than the sunny day. So there are much redundant data which directly obtained from WMS on the sunny days (i.e. the data of rainfall and waterlogging depth are zeros from time (t − m + 1) to (t + d)). Cleaning zero data is the first step of data preprocessing. We employed sliding window to extract the sample at (t − m + 1, t + d). If the waterlogging depth and rainfall from time (t − m + 1) to t is not all 0, the (x(t), x(t-1),…, x(t − m + 1), y(t + d)) will be selected as a sample. In addition, data normalization is also important for waterlogging depth and rainfall. Normalization is a “scaling down” transformation of the features. When the original waterlogging depth and rainfall data are directly used for training and test, the data with higher value is high-lighted and the data with lower value is relatively weakened. Therefore, it is necessary to perform data normalization processing on the extracted feature value to ensure that each feature value is treated equally by the prediction model. In this experiment, z-score normalization was selected to process the original data. The equation of z-score normalization is shown in Eq 13

$$\begin{array}{c} X^{*}\;=\frac{X-\bar{X}}{\sigma } \end{array}$$ (13)

Where $\bar{X}$ and *σ* are the mean value and the standard deviation of the original data, respectively, and X* is the normalized value.

### 4.2 Evaluating measures

In order to assess the effectiveness of the proposed prediction model, three evaluation measures which are MAE, RMSE and R-squared have been introduced MAE is the mean of absolute value of errors. Here, errors are the differences between the predicted values (values predicted by our model) and the actual values. RMSE is the root of the mean of the square of errors. It indicates the absolute fit of the model to the data-how close the actual data points are to the model’s predicted values. The lower values of RMSE indicate better fit. R-squared is a statistical measure that represents the proportion of the variance for a dependent variable that’s explained by an independent variable or variables. It indicates the goodness of fit of the model. R-squared has an useful property that its scale is intuitive: It ranges from zero to one. Zero indicates that the proposed model does not improve prediction over the mean model. One indicates that the prediction is completely same as the actual value. The equation can be expressed as Eqs 14, 15 and 16.

$$\begin{array}{c} MAE=\frac{1}{N}\sum_{i=1}^{N}\left|Y_{act}\left(i\right)-\;Y_{pred}\left(i\right)\right| \end{array}$$ (14)

$$\begin{array}{c} R^{2}=1-\frac{\sum_{i}{\left(Y_{act}\left(i\right)-Y_{pred}\left(i\right)\right)}^{2}}{\sum_{i}{\left(Y_{act}\left(i\right)-\bar{Y}\right)}^{2}} \end{array}$$ (15)

$$\begin{array}{c} RMSE=\sqrt{\frac{\sum_{i=1}^{N}{\left(Y_{act}\left(i\right)-Y_{pred}\left(i\right)\right)}^{2}}{N}} \end{array}$$ (16)

### 4.3 Results and discussion

**Fig 9:**
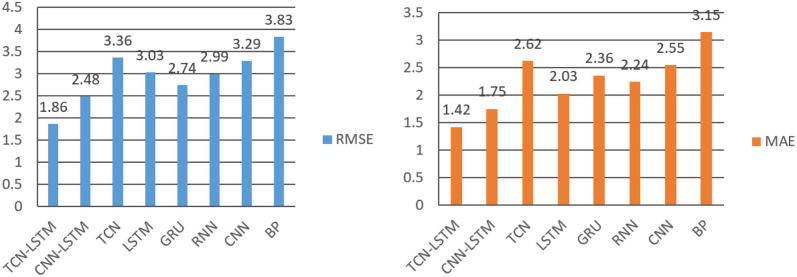
The MAE and RMSE of different waterlogging depth prediction models on HuaiheRoad dataset.

**Fig 10:**
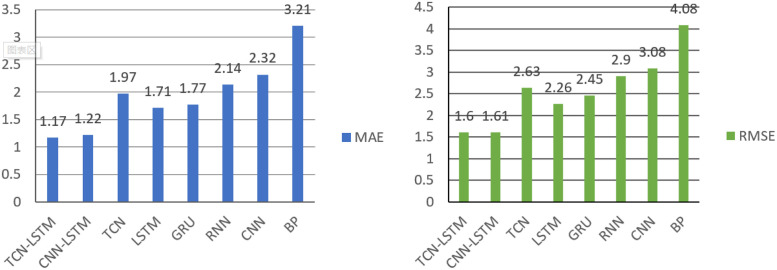
The MAE and RMSE of different waterlogging depth prediction models on DagangzhongRoad dataset.

In this section, we verified the effectiveness of the proposed method by experimental data. First, comparing the TCN-LSTM model with the other seven models (TCN, LSTM, GRU CNN, BP, CNN+LSTM, RNN) on two data sets. Then the TCN-LSTM model was used to predict and visualize the prediction results to further prove the model’s effectiveness. In order to verify the better performance of the combined TCN-LSTM model in predicting waterlogging depth than other models, a few of experiments with LSTM, TCN, RNN, GRU, CNN, BP and CNN-LSTM combined model were also performed. Table 3 shows the results of different prediction models based on three types of evaluation measure, MAE, RMSE and R-squared. The comparison results are also shown in Figs 9 and 10. Due to the difference of model parameters affects the overall prediction results, the model parameters are set consistently in the same data set to ensure the accuracy of model comparison. In this experiment, TCN, RNN, LSTM, GRU and CNN are used as the basic comparison models.

**Table 3:** The MAE, RMSE and R-squared of different models.

Model	Dataset					
	Huaihe Road			Dagangzhong Road		
	MAE	RMSE	R-squared	MAE	RMSE	R-squared
TCN-LSTM	2.0278	3.1948	0.9498	2.0202	3.1029	0.9636
TCN	2.6215	3.3613	0.8757	2.9746	3.6331	0.9255
GRU	2.3662	3.0363	0.9306	2.7703	3.4547	0.9302
RNN	2.2427	2.9934	0.9406	2.1439	3.9084	0.9662
CNN	2.5563	3.2990	0.9224	2.3238	3.0864	0.9516
BP	3.1580	3.8363	0.8671	3.2131	4.0836	0.8596
GBDT	2.4532	3.4133	0.9399	2.4201	3.3118	0.9308
Multi-LSTM	2.6215	3.5613	0.8757	0.8757	3.4331	0.8955
RF-RFE-DNN	3.4662	5.3363	0.8206	3.3703	5.2547	0.8602
RBF-NARX	2.5364	3.1442	0.9159	2.4166	3.1022	0.9258
NB-RF	2.4166	3.1022	0.9258	2.5746	3.4331	0.8955

In order to verify the effectiveness of the proposed model, this section will design a comparison experiment based on the multi-source data set of Ningbo flood disaster constructed in this paper, and conduct experimental comparison with the existing mainstream deep learning prediction model. The comparative experimental data are shown in Table 3.7.

1. GBDT algorithm: Wu [19] et al. proposed GBDT algorithm for the first time to build the prediction model of flood waterlogging point inundation process. Their model trained the sensitive indicators (such as rainfall, rainfall duration, rainfall peak, rainfall peak position coefficient, rainfall intensity variance, etc.) that affect the prediction of flood waterlogging point depth through GBDT algorithm, and achieved certain results.

2. Multi-Step LSTM: Liu et al. [29] introduced the LSTM neural network model into the multi-step prediction model of rainfall and water accumulation at urban waterlogging points. The mean square error (MSE), mean absolute error (MAE) and mean square logarithm error (MSLE) were used as loss functions to train the LSTM model. Three kinds of water accumulation prediction models were constructed, including LSTM (MSE), LSTM (MAE) and LSTM (MSLE), and the multi-step model was used to predict the water accumulation depth in the future 1 h. The measured water data is used to evaluate the prediction results of the model.

3. NB-RF: Wang [30] et al. used naive Bayes (NB) and random forest algorithm (RF) to predict the waterlogging process at waterlogging points and waterlogging points respectively, so as to realize the prediction of the whole process of urban waterlogging. The results show that the prediction results of the NB model are reliable, and the prediction of the RF model is also consistent with the actual situation, which verifies the effectiveness and applicability of the NB model and RF model.

4. RF-RFe-DNN: Bai Lian et al. [31] proposed a flood disaster prediction method for subway stations based on RF-RFE and DNN neural networks. By using random Forest-recursive feature elimination (RF-RFE) algorithm, the importance of initial variables was calculated and the accuracy of variable classification was completed, and important variables were screened from the initial variable set. The DNN neural network prediction model is established, and the selected important variables are taken as input samples to train the DNN neural network to complete the flood disaster prediction of subway stations.

5. RBF-NARX: Wei [20] et al. proposed a neural network model based on the coupled RBF-NARX neural network. Based on the amount of rainfall, an early warning system can predict the whole rainfall process. The results show that the RBF-NARX coupled neural network performs well in the prediction of rainfall and flood level.

Compared with the models of Wu and Liu, the RMSE value of the model proposed in this paper is reduced by 6.4% and 9.3%, respectively. The value of R-squared increased by 3.1% and 9.7% respectively. The reason is likely to be that although the GBDT or Multi-step LSTM model as the water level prediction model can extract part of the features in the data, its feature extraction ability for multi-source data is relatively weak and insufficient, resulting in the final prediction effect still needs to be strengthened. Compared with the model proposed by Bai Lian et al., the model proposed in this paper still has advantages. RMSE values decreased by 4.2% and 4.6%, respectively, in both data sets. The R-square value increased by 5.1% and 5.3%, respectively. The reason may be that the RF-RFE model will screen and sort correlation indicators, and select important indicators from the initial indicators as input samples for the training of DNN network and the final prediction. In this process, some indicators with weak correlation were removed from the sample data due to low ranking, and some data features could not be effectively learned. However, the data set indexes designed in this paper are all sensitive indexes retained after correlation analysis, which may lead to the unsatisfactory actual prediction effect of RF-RFE-DNN model to a certain extent. By contrast, the RBF-NARX coupled neural network proposed by Wei et al. only aims at the modeling of rainfall process and is based on the modeling of historical rainfall and water level in the rainfall process. Compared with the stacked integrated learning model based on multi-source data sets, its data source is too simple, which reduces the prediction accuracy of the model to a certain extent.

**Fig 11:**
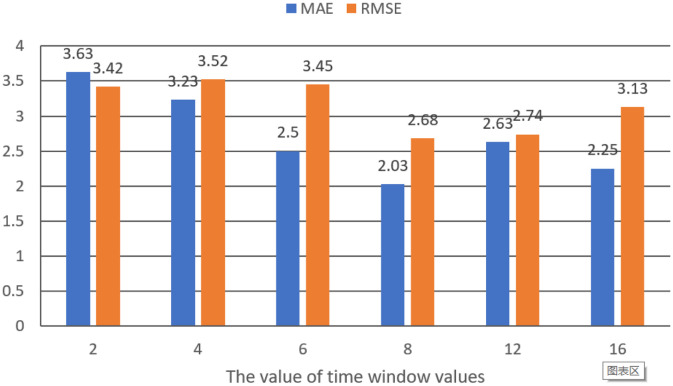
The relationship between the time window size and the effectiveness of prediction model.

The past waterlogging depth and rainfall timely series data is fed to the prediction model. Then the longer the past time frame, the more past waterlogging depth and rainfall timely series data is selected. To study how long historical data TCN-LSTM can be used, we carried out experiments with different historical size of sliding windows to realize the prediction of waterlogging depth. We changed sliding window size from 2, 4, 6, 8, 12 to 16 under the same other conditions. Fig 11 shows the results under different sliding window sizes. It can be seen that with increasing of size of sliding windows, the MAE and RMSE of prediction waterlogging depth fluctuates up and down. The prediction results are better when the time windows values are 8. The experimental results also show that the prediction model after adding the TCN has better effectiveness.

**Fig 12:**
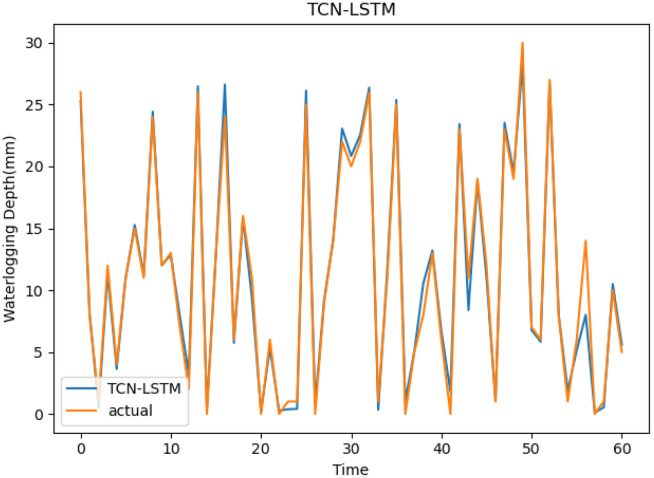
Prediction results of TCN-LSTM model.

**Fig 13:**
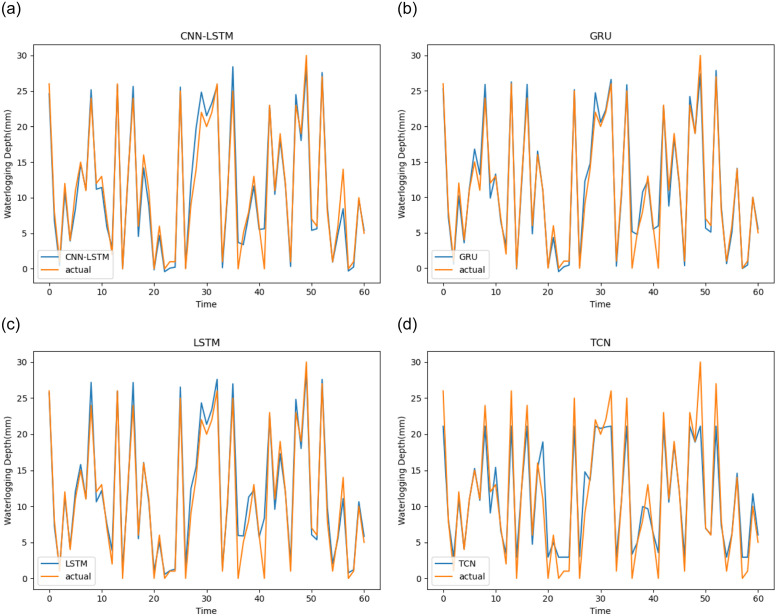
Comparison of prediction results of CNN-LSTM, GRU, LSTM and TCN.

Then we use TCN-LSTM, CNN-LSTM, GRU, LSTM and TCN to predict and visualize the prediction results on Huaihe Road dataset to further prove the proposed model’s effectiveness. It can be observed from Fig 12 that the trend of the prediction curve is almost the same as that of the waterlogging depth truth curve. Fig 13(b)–13(d) shows the predicted values of TCN, LSTM, and GRU, and the actual ones. From Fig 13, it can be found that the accuracy of three models is much closer, and at most slots, the predicted value of LSTM are closer to the actual value than other two models. Through the comparison of Figs 12 and 13, the curve fitting degree between the predicted value of TCN-LSTM and the actual value of waterlogging depth is the highest. At most time slots, the predicted value of TCN-LSTM is closer to the actual one than other methods, thereby proving its effectiveness. Fig 13(d) shows the predicted values of CNN-LSTM, and the actual values, from which it can be found that the accuracy is much lower than TCN-LSTM model. This shows that it is unwise to use standard CNN to replace TCN. In conclusion, our proposed TCN-LSTM achieves the highest prediction accuracy.

In general, WMS acquires data timely, accurately and conveniently, and the acquired data is time series sequence data. In order to be able to effectively extract effective information from long sequence data, we propose the TCN-LSTM network model. TCN has more flexible receptive field size and LSTM has the ability to effectively extract the dependencies of time series data. The results show that the proposed model is better than the conventional RNN model for time series data processing, such as LSTM model, GRU model, etc. Compared with the hydrological model and image detection model, the data acquisition is convenient and real-time by WMS, the TCN-LSTM model has the ability to predict timely. This is also consistent with our original intention. We hope to establish an effective urban waterlogging depth prediction model, which has low cost, easy data acquisition, and can predict timely and accurately. Overall, the low-cost method proposed in this study can be used to obtain timely waterlogging warning information, and enhance the possibility of using existing social networks and traffic surveillance video systems to perform opportunistic waterlogging sensing.

## 5 Conclusion

Because of the growing problem of urban waterlogging disasters, this paper introduces a method of predicting urban waterlogging depth based on the TCN-LSTM model and WMS. In the proposed method, WMS is used to obtain urban waterlogging depth and rainfall timely series data. TCN-LSTM model is used to predict waterlogging depth based on the past rainfall and waterlogging depth series data. In order to assess the effectiveness of the proposed method, we conducted some experiments to verify the effectiveness of different prediction models. The experimental results show that MAE and RMSE of the TCN-LSTM model are lower than other deep learning models, such as LSTM, RNN, CNN, GRU and CNN-LSTM models. Compared with the hydrological model and image detection model, the data acquisition is convenient and real-time by WMS, the TCN-LSTM model has the ability to predict timely. Therefore, it is very effective for stacking up TCN-LSTM model to predict the waterlogging depth and using WMS to collect data. Overall, the low-cost method proposed in this study can be used to obtain timely waterlogging warning information, and enhance the possibility of using existing social networks and traffic surveillance video systems to perform opportunistic waterlogging sensing.

In addition, the authors attempt to study more efficient features and structures to promote the model prediction ability, such as, studying the impact of near regions’ waterlogging depth to one location prediction. Since the distance between different regions is relatively short, the change of the waterlogging depth in one region is likely to affect another region.

## Supporting information

S1 Data (BIB)
